# Lymphocytic colitis presenting as difficult diarrhoea in an African woman: a case report and review of the literature

**DOI:** 10.1186/1752-1947-4-31

**Published:** 2010-01-29

**Authors:** Udeme E Ekrikpo, Jesse A Otegbayo, Abideen O Oluwasola

**Affiliations:** 1 Department of Medicine, University College Hospital, PMB 5116, Ibadan, Nigeria; 2 Department of Pathology, University College Hospital, PMB 5116, Ibadan, Nigeria

## Abstract

**Introduction:**

Lymphocytic colitis is an uncommon intestinal disorder that presents with chronic diarrhoea. It is treatable, but in the developing world, its diagnosis may often prove difficult. Data and reports of this condition in Africa are scarce because most medical centres lack a functional gastrointestinal endoscopy unit that would aid in the diagnosis.

**Case presentation:**

We present the case of a 53-year-old Nigerian woman with pathogen-negative chronic diarrhoea and a family history of chronic diarrhoea. She responded well to treatment after colonoscopy and colonic biopsy successfully diagnosed her illness.

**Conclusion:**

Referral of patients with pathogen-negative chronic diarrhoea to medical centres that have facilities for colonoscopy and biopsy is important in the developing world.

## Introduction

In the developing world where there is scarcity of facilities for endoscopy in many medical centres, patients presenting with chronic or recurrent diarrhoea for which no infective, metabolic or mechanical cause is found are usually thought to have the diarrhoeal type of irritable bowel syndrome and therefore managed empirically as such.

Lymphocytic colitis and collagenous colitis make up a group of uncommon large bowel inflammatory conditions called microscopic colitis. It is yet to be fully ascertained if these two clinical conditions are separate entities, albeit with similar clinical presentation, or if they are clinical manifestations of a spectrum of clinical conditions [1]. The implication of certain drugs such as ranitidine, ticlopidine, flutamide, carbamazepine, sertraline, paroxetine, simvastatin and Cyclo 3 Fort in the aetiopathogenesis makes the clinical picture more complex [2-7]. There is a lack of information regarding these conditions in Africa. Information on prevalence, clinical features, clinical course and response to therapy is not well documented in the continent and had been limited to case reports [8]. Microscopic colitides are potentially treatable if a high index of suspicion is maintained and facilities are available for endoscopy and histological diagnosis. We present a case of lymphocytic colitis in an African woman.

## Case presentation

A 53-year-old Nigerian woman with a 10-year history of recurrent passage of loose watery stools was referred to our facility following several unsuccessful antidiarrhoeal therapies and a suspicion of colonic tumour. She had five to six episodes daily of watery, non-mucoid and non-bloody stools not associated with vomiting, abdominal pain or cramps. There was no weight loss or history of passage of undigested food particles and there were no features of fluid retention. There was also no history of joint pains and swelling or use of non-steroidal anti-inflammatory drugs (NSAIDs). Physical examination did not reveal any abnormality except for bradycardia (48-56/min). There was a positive family history of chronic diarrhoea in the elder sister.

The laboratory investigation revealed no ova or cyst of parasitic origin in the stools, and the stool culture yielded no pathogens. Complete blood count, liver function tests, erythrocyte sedimentation rate, lipid profile, and serum electrolytes, urea and creatinine levels were within the normal range. The HIV screening was non-reactive.

Serum amylase was raised at 557 ug/l (normal range: 25-125 ug/l), while serum lipase was normal at 33 ug/l (normal range: 25-57 ug/l).

After bowel preparation, she underwent fibreoptic colonoscopy with random biopsies taken at the ascending and descending colons after no mass lesion or inflammation were found.

**Figure 1:**
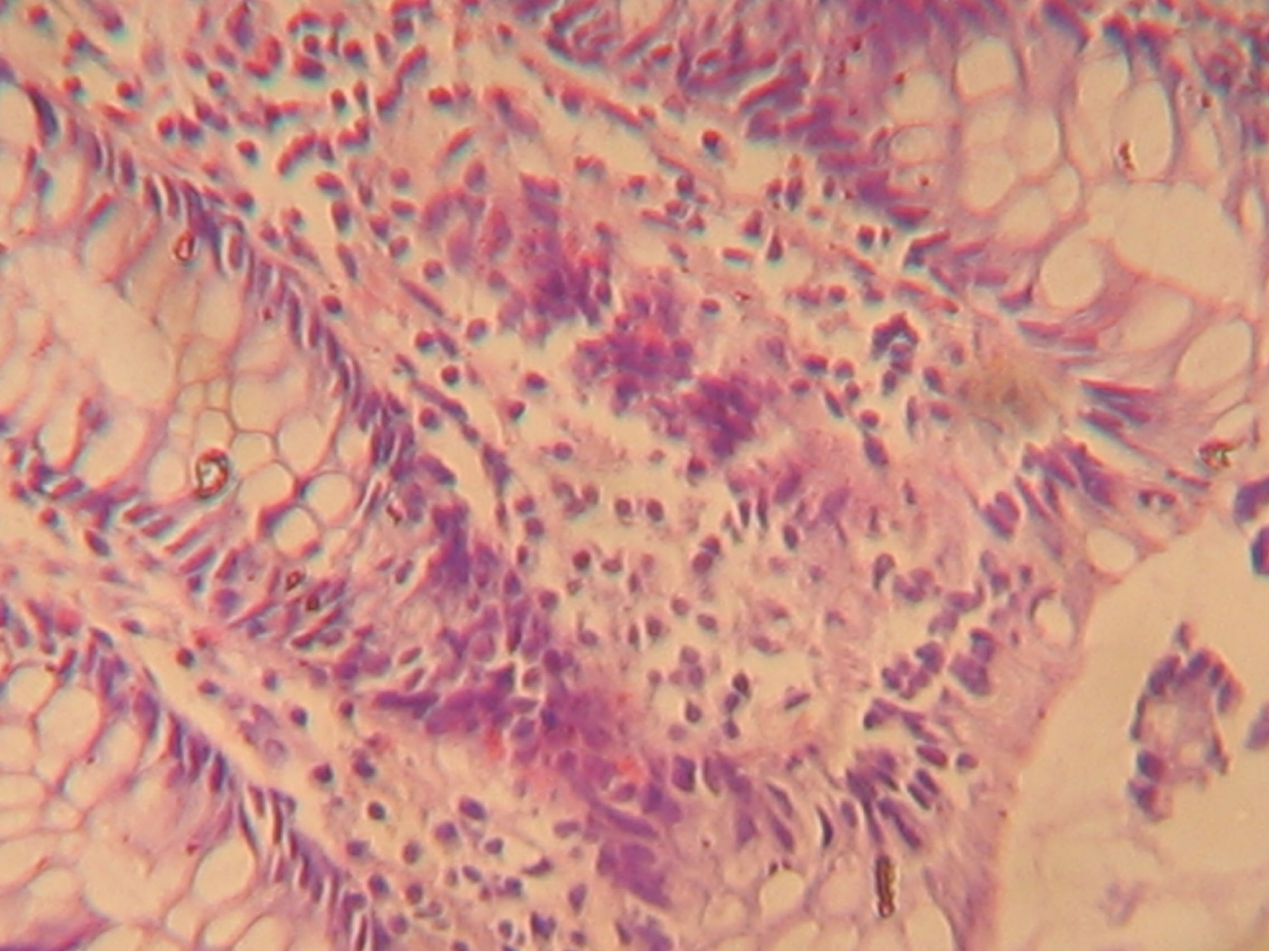
**A high power micrograph of colonic mucosa showing moderate mucosal chronic inflammation and focal intraepithelial infiltrates of lymphocytes**.

The histopathology report of the colonic biopsy showed benign surface columnar epithelium admixed with goblet cells and many simple glands lined by a layer of goblet cells within the lamina propria. There was moderate infiltrate of a mixed population of chronic inflammatory cells within a mildly oedematous lamina propria, consisting of lymphocytes and plasma cells with a focus of lymphoid follicle formation. There were also few eosinophils and neutrophil polymorphs, with focal intraepithelial infiltration by lymphocytes. These features were in keeping lymphocytic colitis (Figure 1).

The patient was subsequently placed on bismuth subsalicylate. This resulted to the complete resolution of the diarrhoea after about two months of therapy. She is still being followed up in clinic.

## Discussion

Lymphocytic colitis (LC) and collagenous colitis (CC) belong to the group of microscopic colitides, a term which was first introduced by Read *et al. *in 1980 [9]. LC was first described by Lazenby *et al. *in 1989 [10] to replace the term microscopic colitis and to distinguish it from infectious colitis and inflammatory bowel disease (ulcerative colitis and Crohn's disease). Earlier, Lindstrom had described CC [11]. Some, however, view the two as related but distinct syndromes [12]. LC and CC are relatively rare conditions diagnosed when a patient with chronic, watery and non-bloody diarrhoea has an endoscopically or radiographically normal colon, but colonic biopsies show unique inflammatory changes. Because the mucosa is not ulcerated or otherwise disrupted, the diarrhoea generally does not contain blood or pus [13].

LC has not been extensively studied in many populations. Its true incidence in the USA is not known [13]. A study in Iceland put the mean annual incidence of LC at four per 100,000 inhabitants in the period 1995 to1999 [14]. LC shows no gender predilection, whereas CC is about 20 times more common in women than in men [13]. Bohr *et al*. [15] recorded an annual incidence of 4.4 out of 100,000 female inhabitants and 3.0 out of 100,000 male inhabitants in a Swedish population. Our patient was 52 years old and falls within the typical age at diagnosis, which is 50 to 72 years in women [1]. Our patient presented with the classical features of LC which include chronic or recurrent non-bloody, non-mucoid diarrhoea with infrequent occurrence of abdominal cramps, faecal incontinence and weight loss.

No definite aetiology has been determined for LC. Nonetheless, many case reports describe patients with pre-existing, presumed autoimmune conditions, such as celiac sprue and rheumatoid arthritis, who subsequently are diagnosed with LC. Some patients diagnosed with LC also had concurrent uveitis, idiopathic pulmonary fibrosis, juvenile diabetes mellitus, pernicious anaemia, autoimmune thyroid disease, and idiopathic thrombocytopenic purpura. The coincidence of celiac sprue and LC raises the possibility of a luminal agent being responsible for the colitis; however, removal of gluten from the diet is ineffective in treating the colitis. A family history of chronic diarrhoea may be important in some individuals as it was in our patient, which may indicate a genetic predisposition in this condition. In a Swedish study [15], familial occurrence was discovered in five families and there was a sister/sister relationship in these patients as was also noted in our patient. Another suspected aetiology is exposure to certain drugs though this was not observed in our patient.

The absence of macroscopic lesions on colonoscopy in our patient is characteristic of lymphocytic colitis. Biopsy specimens taken in other patients show, on histopathologic examination, intraepithelial lymphocytes >20 per 100 surface epithelial cells; epithelial damage, for example, flattening and mucin depletion; inflammation in the lamina propria with mainly mononuclear cells; and a sub-epithelial collagen layer <10 um [4]. These were also the dominant histological findings in our patient who is of African descent, thus suggesting no significant histological differences between findings when compared with Caucasian patients. This is important as most cases of diarrhoea in Africans are caused bacterial infections and parasitic infestation. More recently, confocal endomicroscopy has been used to make diagnosis during colonoscopy, but this is presently only available at a few medical centres.

A number of drugs have been implicated in the aetiopathogenesis of lymphocytic colitis. These include carbamazepine, sertraline, paroxetine, ticlopidine and simvastatin. Drugs such as ranitidine and Cyclo-3-Fort have also been implicated, and NSAIDs may also be causative. No drug could be implicated in our patient. The differential diagnoses of LC include coeliac sprue, inflammatory bowel disease, irritable bowel syndrome, thyrotoxicosis and giardiasis. None of these fit the presentation in our patient.

Sulfasalazine, mesalamine, bismuth subsalicylate, cholestyramine, loperamide, diphenoxylate hydrochloride and atropine sulphate have all been used with variable results in treating LC. Steroids have also been tried in cases where the regular drugs do not give the desired relief. Methotrexate and azathioprine are usually reserved for the few cases that do not respond to steroids or any of the abovementioned drugs. Our patient responded well to bismuth subsalicylate. Recently, a double-blind, randomized placebo-controlled trial showed significant effectiveness of budesonide in the treatment of CC [16]. LC runs a benign course and most of the patients achieve symptomatic and histopathologic resolution within months of treatment. A repeat colonoscopy and biopsy is important for these patients.

## Conclusions

Lymphocytic colitis and collagenous colitis exist in the African population. In less-developed economies where there is a lack of endoscopic facilities, efforts should be made to ensure colonoscopy and biopsies for all patients with watery diarrhoea when the common infective causes have been excluded. This will help clinicians recognize a potentially treatable condition and allow institution of adequate treatment measures.

## Consent

Written informed consent was obtained from the patient for publication of this case report and any accompanying images. A copy of the written consent is available for review by the Editor-in-Chief of this journal.

## Abbreviations

CC: collagenous colitis; LC: lymphocytic colitis; NSAIDs: non-steroidal anti-inflammatory drugs.

## Competing interests

The authors declare that they have no competing interests.

## Authors' contributions

JA performed the initial assessment, colonoscopy and biopsy of the patient. AO performed the histologic examination of the colonic biopsy specimen; while UE played a major role in writing the manuscript and follow up of the patient. All authors read and approved the final manuscript.
